# Association Between Maximum Temperature 24 Hours After Resuscitation and Survival for In‐Hospital Cardiac Arrest

**DOI:** 10.1161/JAHA.125.048549

**Published:** 2026-08-20

**Authors:** Omar Cantu‐Martinez, Mohammad Abdel Jawad, Kevin F. Kennedy, Andrew A. Girard, Nobuhiro Ikemura, John A. Spertus, Paul S. Chan, for the American Heart Association's Get With The Guidelines–Resuscitation Investigators, Anne Grossestreuer, Ari Moskowitz, Joseph Ornato, Matthew Churpek, Paul Chan, Saket Girotra, Sarah Perman

**Affiliations:** ^1^ Saint Luke's Mid America Heart Institute Kansas City MO USA; ^2^ University of Missouri–Kansas City's Healthcare Institute for Innovations in Quality Kansas City MO USA; ^3^ Department of Cardiology Keio University School of Medicine Tokyo Japan

**Keywords:** cardiac arrest, fever, postresuscitation care, temperature, Cardiopulmonary Arrest, Cardiopulmonary Resuscitation and Emergency Cardiac Care, Mortality/Survival

## Abstract

**Background:**

Avoiding fever is a central tenet of postresuscitation care. However, few data exist describing the association between postresuscitation temperature and survival after in‐hospital cardiac arrest, where response is usually prompt.

**Methods:**

Within the GWTG–Resuscitation (Get With The Guidelines–Resuscitation) registry, we identified adults from 2015 to 2024 with an index in‐hospital cardiac arrest and return of spontaneous circulation who survived at least 24 hours after resuscitation. Patients were excluded if they received therapeutic hypothermia after return of spontaneous circulation to better understand the natural history of postresuscitation temperature. The primary exposure was maximum temperature 24 hours after return of spontaneous circulation, categorized by 0.5 °C intervals from <36.3 °C to ≥38.8 °C. Multivariable hierarchical logistic regression evaluated the association between maximum temperature and both survival to hospital discharge and favorable neurological survival.

**Results:**

Of 36 514 with return of spontaneous circulation, mean age was 64.2±16.6 years; 61.8% were men, and 69.4% were White individuals. Median maximum temperature was 37.2 °C (interquartile range, 36.8–37.8 °C), 66.3% survived to discharge, and 57.0% had favorable neurological survival. The highest survival (72.7%) was observed in the 36.8 to <37.3°C group (reference), with all other temperature ranges with lower rates of survival to hospital discharge (inverted U‐shaped relationship), with the worst being those in the lowest (<36.3 °C, 48.4%; adjusted odds ratio, 0.42 [95% CI, 0.37–0.46]) and highest (≥38.8 °C, 51.5%; adjusted odds ratio, 0.40 [95% CI, 0.36–0.44]) ranges. A similar relationship was observed with favorable neurological survival.

**Conclusions:**

Among patients not treated with therapeutic hypothermia, a postresuscitation temperature of 36.8 to 37.3°C after in‐hospital cardiac arrest was associated with the highest survival rate, with temperatures at high and low extremes with the lowest survival.

**Nonstandard Abbreviations and Acronyms**
GWTG–Resuscitation: Get With The Guidelines–Resuscitation
IHCA: in‐hospital cardiac arrest
OHCA: out‐of‐hospital cardiac arrest
ROSC: return of spontaneous circulation

**Clinical Perspectives**

**What Is New?**

- The association between postresuscitation temperature and survival after in‐hospital cardiac arrest has not been well described.
- In a large national registry of contemporary patients with in‐hospital cardiac arrest not treated with therapeutic hypothermia, we found an inverted U‐shaped relationship between maximum temperature during the first 24 hours after return of spontaneous circulation and survival outcomes.

**What Are the Clinical Implications?**

- The highest survival occurred among patients with a maximum temperature between 36.8 and <37.3 °C. Both lower (<36.3 °C) and higher (≥38.8 °C) maximum temperatures were associated with substantially worse survival and neurological outcomes.

Avoiding fever after cardiac arrest is a central tenet of postresuscitation care. However, existing data on postresuscitation temperature and survival are >2 decades old, from studies with small sample sizes, and largely from patients with out‐of‐hospital cardiac arrest (OHCA).^1^, ^2^, ^3^, ^4^, ^5^ As these studies found that patients with higher postresuscitation temperatures had worse survival outcomes, current postresuscitation guidelines recommend fever prevention as critical to improving survival and neurological outcomes.^6^

For in‐hospital cardiac arrest (IHCA), cardiopulmonary resuscitation is typically started almost immediately,^7^ as compared with a median of 8 to 10 minutes for OHCA.^8^, ^9^ Consequently, patients with IHCA have a shorter no‐flow circulatory state, which may result in less anoxic brain injury. Moreover, patients with IHCA differ from those with OHCA by having potentially reversible acute medical concerns precipitating their arrest and more likely having a nonshockable rhythm. Therefore, there may be a different relationship between postresuscitation temperature and survival with IHCA, as compared with OHCA. Because most patients successfully resuscitated from IHCA do not receive therapeutic hypothermia in routine practice,^10^ there is a need to better understand the natural history between postresuscitation temperature and survival outcomes in this population. To address this need, we examined the relationship between maximum temperature during the first 24 hours after return of spontaneous circulation (ROSC) and survival outcomes in a large national IHCA registry.

## Methods

### Data Source

Because of the sensitive nature of the data collected for this study, requests to access the data set from qualified researchers trained in human subject confidentiality protocols may be sent to the American Heart Association at qualitynow@heart.org. This study leveraged data from the GWTG–Resuscitation (Get With The Guidelines–Resuscitation) registry, a prospective, hospital‐based quality improvement program sponsored by the American Heart Association that collects detailed information on IHCA across US hospitals. Participation in the registry is voluntary, and its design and data collection methods have been previously described.^11^ Hospitals participating in the registry through certified hospital staff submit clinical information about the medical history, hospital care, and outcomes of consecutive patients hospitalized for cardiac arrest, excluding those with do‐not‐resuscitate orders, using an online, interactive case report form and Patient Management Tool (IQVIA, Parsippany, NJ). IQVIA serves as the data collection (through their Patient Management Tool) and coordination center for the American Heart Association/American Stroke Association Get With The Guidelines programs. Cases are identified through multiple methods, including centralized collection of cardiac arrest flow sheets, reviews of hospital paging system logs, and routine checks of code carts and pharmacy tracer drug records. Standardized “Utstein‐style” definitions for patient variables and outcomes are used to ensure uniform reporting across hospitals.^12^ Institutional review board approval was granted and informed consent was waived as this research was not classified as human‐subject research and involved only deidentified data.

### Study Population

We identified adult patients with an index IHCA who achieved ROSC between 2015 and 2024. We excluded patients who experienced an initial OHCA before their IHCA and those with prearrest sepsis, COVID‐19, or pneumonia, as these conditions could independently cause fever. Patients treated with therapeutic hypothermia after ROSC were also excluded, to better understand the natural history of postresuscitation temperature after IHCA and as the GWTG–Resuscitation registry did not collect data to determine whether hypothermia was initiated to prevent fever or in response to it. Because the variable of interest was maximum temperature within the first 24 hours after ROSC, patients who died during that period were excluded. Finally, we excluded patients with a maximum temperature <30 or >44 °C, as these represented extreme outliers likely due to documentation errors. Patients from hospitals with >25% missing temperature data, as well as patients from hospitals below this threshold who were missing maximum temperature data, were also excluded. Our final study cohort included 36 514 patients from 345 hospitals who achieved ROSC after IHCA.

### Independent Variable and Study Outcome

The independent variable was the maximum recorded temperature within the first 24 hours after ROSC, categorized in 0.5 °C intervals: <36.3 °C, 36.3 to <36.8 °C, 36.8 to <37.3 °C, 37.3 to <37.8 °C, 37.8 to <38.3 °C, 38.3 to <38.8 °C, and ≥38.8 °C. The primary outcome was survival to hospital discharge, and the secondary outcome was favorable neurological survival, defined as survival with a discharge Cerebral Performance Category score (range, 1–5) of 1 (denoting mild to no neurological disability) or 2 (denoting moderate neurological disability).

### Statistical Analysis

Baseline characteristics of patients across temperature groups were compared using 1‐way ANOVA for normally distributed continuous variables and the Kruskal–Wallis test for continuous variables with nonnormally distributions. Categorical variables were compared using the χ^2^ test. To assess potential selection bias, baseline characteristics were compared between patients included in the final study cohort and those excluded because of missing temperature data. Differences between groups were summarized using standardized mean differences (SMD), with SMD values ≥0.25 considered indicative of meaningful imbalance.^13^

To examine the association between maximum temperature during the first 24 hours after ROSC and survival to hospital discharge, we constructed a 2‐level multivariable hierarchical logistic regression model, with hospitals treated as a random effect to account for clustering of patient outcomes within sites. Maximum postresuscitation temperature was modeled primarily as a categorical variable as described above, with 36.8 to <37.3 °C as the reference group, as this range includes normal physiologic body temperature. To further characterize the association across the full temperature range, we also modeled maximum temperature as a continuous variable using restricted cubic splines with 3 knots to estimate and plot predicted probabilities of survival to discharge with 95% CIs. To mitigate potential confounding, the model adjusted for clinically relevant patient‐, arrest‐, and intervention‐related covariates that could be associated with both temperature and outcomes as fixed effects: age, sex, race and ethnicity, initial arrest rhythm (asystole, pulseless electrical activity, ventricular fibrillation, or pulseless ventricular tachycardia), location of arrest, illness category, and comorbid conditions and preexisting conditions before IHCA (history of or index admission for myocardial infarction; history of or index admission for heart failure, diabetes, renal insufficiency, hepatic insufficiency, respiratory insufficiency, hypotension, acute stroke, baseline central nervous system depression, metastatic or hematologic malignancy, and metabolic or electrolyte abnormalities). Interventions in place at the time of cardiac arrest, including mechanical ventilation, hemodialysis, and continuous intravenous vasopressors, were also included as covariates.

We then repeated the above analyses to examine the association between postresuscitation temperature and the secondary outcome, favorable neurological survival. Since the discharge Cerebral Performance Category score is used to define favorable neurological survival and was missing for 2955 (8.1%) of all patients, we performed multiple imputation based on all available patient data to estimate the missing score in these patients. We repeated the analyses on each imputed data set and pooled the results using Rubin's method to obtain final effect estimates.^14^

To evaluate whether the association between maximum temperature and survival was consistent among patients who were likely comatose after ROSC, we performed a sensitivity analysis restricted to patients who received ≥5 minutes of cardiopulmonary resuscitation before achieving ROSC. The primary analysis included patients with any cardiopulmonary resuscitation duration. This sensitivity analysis repeated the 2‐level multivariable hierarchical logistic regression models for survival to hospital discharge and favorable neurological survival, as described above.

All analyses were assessed using a 2‐sided α of 0.05 and were completed with SAS version 9.4 (SAS Institute Inc., Cary, NC, USA).

## Results

### Patient Characteristics

Among 174 821 patients with an index IHCA, 36514 patients who achieved ROSC, survived for ≥24 hours, and had available temperature data were included in the final study cohort (Figure 1). Excluded patients included 17 669 with an initial OHCA before their IHCA; 34394 who died within 24 hours; and 48 008 with sepsis, COVID‐19 infection, or pneumonia. To assess potential selection bias, demographic‐, clinical‐, and arrest‐related characteristics were compared between included patients and those excluded because of missing temperature data (Tables S1). Included and excluded patients generally had similar characteristics, with most SMDs <0.10 and all SMDs <0.20.

**Figure 1:**
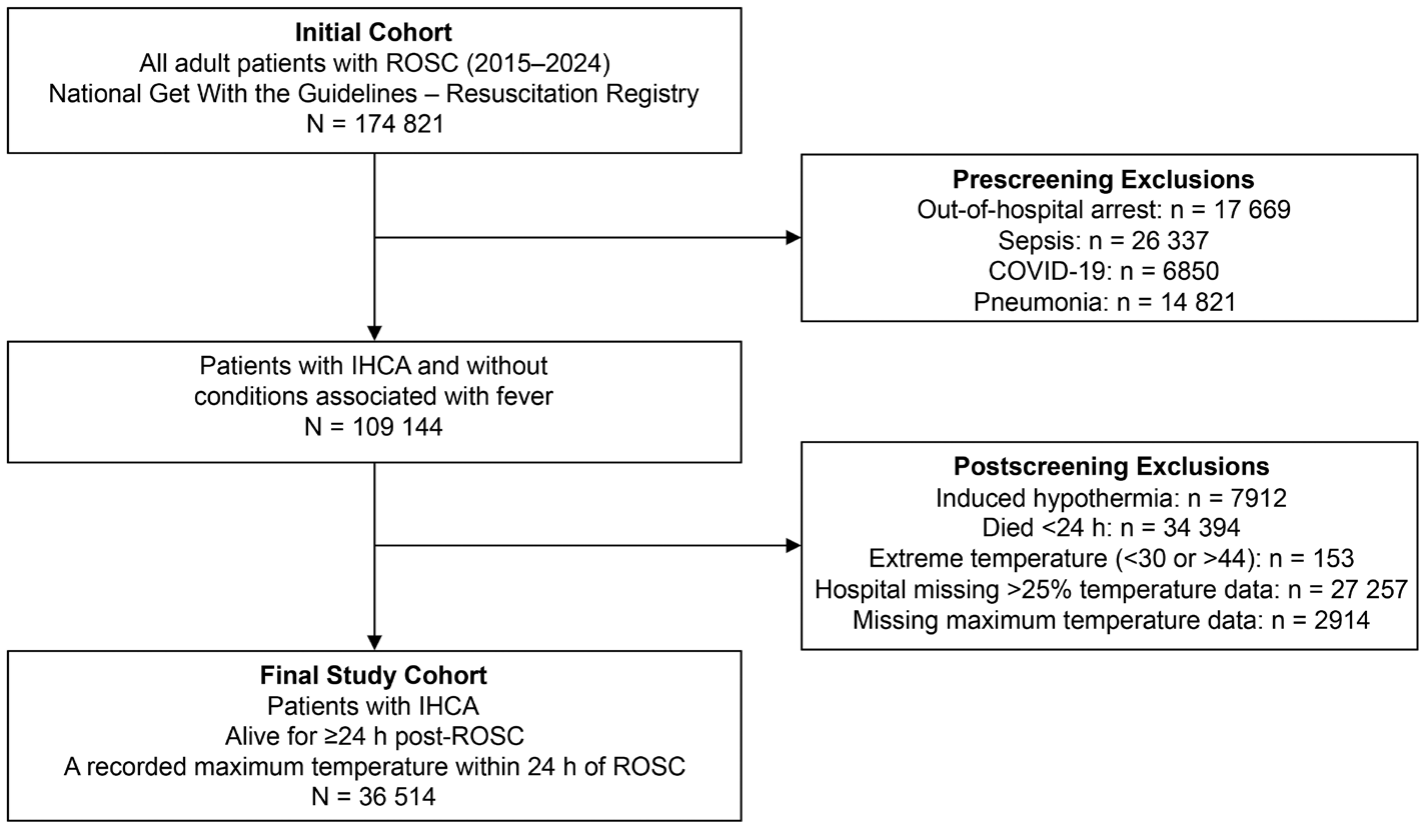
Flowchart of the study cohort. Shows the initial cohort and the final analytic study cohort after applying exclusion criteria. IHCA indicates in‐hospital cardiac arrest; and ROSC, return of spontaneous circulation.

Mean age in the study cohort was 64.2±16.6 years, and 61.8% were men (Table 1). Most patients were White individuals (69.4%), 21.0% were Black individuals, 2.2% were Asian individuals, and 7.4% were of other racial groups or unknown race. The most prevalent preexisting conditions at the time of cardiac arrest were respiratory insufficiency (40.0%), diabetes (38.3%), renal insufficiency (30.5%), and hypotension (24.6%). Most patients had an initially nonshockable arrest rhythm (pulseless electrical activity [50.4%] and asystole [22.6%]) whereas 27.0% had a shockable rhythm. Nearly 2 in 5 patients had their IHCA in the intensive care unit, and >72% had a medical admission diagnosis. For interventions in place at the time of cardiac arrest, 22.2% were on mechanical ventilation, 19.3% were on a continuous vasopressor infusion, and 3.0% were on dialysis.

**Table 1:** Patient Characteristics Stratified by Maximum Temperature During the First 24 Hours After ROSC

Characteristics	Overall	<36.3 °C	36.3 to <36.8 °C	36.8 to <37.3 °C	37.3 to <37.8 °C	37.8 to <38.3 °C	38.3 to <38.8 °C	≥38.8 °C	*P* value
	n=36 514	n=2023	n=5770	n=11 637	n=7618	n=4790	n=2524	n=2152	
Maximum temperature, °C, median (IQR)	37.2 (36.8–37.8)	35.9 (35.4–36.1)	36.6 (36.5–36.7)	37 (36.9–37.2)	37.5 (37.4–37.7)	38 (37.9–38.1)	38.5 (38.4–38.6)	39.2 (39.0–39.5)	<0.001
Demographics									
Age, y, mean±SD	64.2±16.6	64.1±20.1	66.1±14.4	65.4±15.6	64.4±17.7	63.3±17.5	61.4±15.7	58.0±16.5	<0.001
Sex, n (%)									<0.001
Male	22 583 (61.8)	1200 (59.3)	3545 (61.4)	7119 (61.2)	4679 (61.4)	3008 (62.8)	1592 (63.1)	1440 (66.9)	
Female	13 931 (38.2)	823 (40.7)	2225 (38.6)	4518 (38.8)	2939 (38.6)	1782 (37.2)	932 (36.9)	712 (33.1)	
Race and ethnicity, n (%)									<0.001
White	25 330 (69.4)	1224 (60.6)	3936 (68.2)	8250 (70.9)	5278 (69.3)	3364 (70.2)	1767 (70.0)	1511 (70.2)	
Black	7681 (21.0)	547 (27.1)	1312 (22.7)	2337 (20.1)	1601 (21.0)	991 (20.7)	477 (18.9)	416 (19.3)	
Asian non‐Hispanic	792 (2.2)	55 (2.7)	127 (2.2)	221 (1.9)	176 (2.3)	93 (1.9)	75 (3.0)	45 (2.1)	
Other or unknown	2709 (7.4)	195 (9.6)	395 (6.8)	829 (7.1)	563 (7.4)	342 (7.1)	205 (8.1)	180 (8.4)	
Hispanic	2225 (6.1)	135 (6.7)	314 (5.4)	677 (5.8)	459 (6.0)	287 (6.0)	181 (7.2)	172 (8.0)	
Preexisting conditions, n (%)									
Respiratory insufficiency	14 621 (40.0)	826 (40.8)	1933 (33.5)	4044 (34.8)	3227 (42.4)	2284 (47.7)	1207 (47.8)	1100 (51.1)	<0.001
Diabetes	13 995 (38.3)	754 (37.3)	2380 (41.2)	4441 (38.2)	2872 (37.7)	1773 (37.0)	966 (38.3)	809 (37.6)	<0.001
Renal insufficiency	11 123 (30.5)	805 (39.8)	2014 (34.9)	3550 (30.5)	2252 (29.6)	1272 (26.6)	654 (25.9)	576 (26.8)	<0.001
Hypotension	8977 (24.6)	541 (26.7)	1176 (20.4)	2589 (22.2)	2029 (26.6)	1340 (28.0)	713 (28.2)	589 (27.4)	<0.001
Metabolic or electrolyte abnormality	7311 (20.0)	573 (28.3)	1078 (18.7)	2037 (17.5)	1558 (20.5)	1047 (21.9)	543 (21.5)	475 (22.1)	<0.001
Heart failure this admission	5647 (15.5)	297 (14.7)	985 (17.1)	1903 (16.4)	1161 (15.2)	683 (14.3)	325 (12.9)	293 (13.6)	<0.001
History of prior heart failure	10 022 (27.4)	551 (27.2)	1843 (31.9)	3317 (28.5)	2011 (26.4)	1264 (26.4)	561 (22.2)	475 (22.1)	<0.001
Myocardial infarction this admission	6299 (17.3)	327 (16.2)	1046 (18.1)	2207 (19.0)	1275 (16.7)	771 (16.1)	395 (15.6)	278 (12.9)	<0.001
History of myocardial infarction	5956 (16.3)	346 (17.1)	1072 (18.6)	2041 (17.5)	1176 (15.4)	722 (15.1)	341 (13.5)	258 (12.0)	<0.001
Acute central nervous system nonstroke event	4372 (12.0)	216 (10.7)	614 (10.6)	1298 (11.2)	966 (12.7)	600 (12.5)	371 (14.7)	307 (14.3)	<0.001
Acute stroke	1537 (4.2)	65 (3.2)	212 (3.7)	430 (3.7)	333 (4.4)	241 (5.0)	125 (5.0)	131 (6.1)	<0.001
Baseline depression in central nervous system function	2505 (6.9)	136 (6.7)	365 (6.3)	726 (6.2)	540 (7.1)	368 (7.7)	189 (7.5)	181 (8.4)	<0.001
Metastatic or hematologic malignancy	3163 (8.7)	211 (10.4)	480 (8.3)	955 (8.2)	662 (8.7)	425 (8.9)	232 (9.2)	198 (9.2)	0.03
Hepatic insufficiency	2579 (7.1)	226 (11.2)	446 (7.7)	757 (6.5)	531 (7.0)	297 (6.2)	166 (6.6)	156 (7.2)	<0.001
Major trauma	1996 (5.5)	67 (3.3)	149 (2.6)	393 (3.4)	460 (6.0)	385 (8.0)	256 (10.1)	286 (13.3)	<0.001
Cardiac arrest characteristics, n (%)									
Initial arrest rhythm									<0.001
Asystole	8248 (22.6)	479 (23.7)	1253 (21.7)	2635 (22.6)	1739 (22.8)	1029 (21.5)	564 (22.3)	549 (25.5)	
Pulseless electrical activity	18 416 (50.4)	1166 (57.6)	2748 (47.6)	5351 (46.0)	3936 (51.7)	2666 (55.7)	1390 (55.1)	1159 (53.9)	
Ventricular fibrillation	5361 (14.7)	214 (10.6)	930 (16.1)	2032 (17.5)	1079 (14.2)	579 (12.1)	294 (11.6)	233 (10.8)	
Pulseless ventricular tachycardia	4489 (12.3)	164 (8.1)	839 (14.5)	1619 (13.9)	864 (11.3)	516 (10.8)	276 (10.9)	211 (9.8)	
Location of cardiac arrest									<0.001
Intensive care unit	13 936 (38.2)	777 (38.4)	1890 (32.8)	3982 (34.2)	3034 (39.8)	2072 (43.3)	1174 (46.5)	1007 (46.8)	
Telemetry	6416 (17.6)	297 (14.7)	1202 (20.8)	2324 (20.0)	1276 (16.7)	666 (13.9)	351 (13.9)	300 (13.9)	
Nonmonitored	4955 (13.6)	311 (15.4)	861 (14.9)	1525 (13.1)	1031 (13.5)	659 (13.8)	288 (11.4)	280 (13.0)	
Emergency room	4983 (13.6)	340 (16.8)	720 (12.5)	1514 (13.0)	1023 (13.4)	693 (14.5)	370 (14.7)	323 (15.0)	
Procedural	5404 (14.8)	242 (12.0)	949 (16.4)	2002 (17.2)	1102 (14.5)	615 (12.8)	294 (11.6)	200 (9.3)	
Other	818 (2.2)	56 (2.8)	148 (2.6)	288 (2.5)	152 (2.0)	85 (1.8)	47 (1.9)	42 (2.0)	
Illness category									<0.001
Medical, cardiac	13 547 (37.1)	750 (37.1)	2551 (44.2)	4829 (41.5)	2593 (34.0)	1458 (30.4)	769 (30.5)	597 (27.7)	
Medical, noncardiac	12 905 (35.3)	853 (42.2)	2026 (35.1)	3801 (32.7)	2757 (36.2)	1699 (35.5)	901 (35.7)	868 (40.3)	
Surgical, cardiac	3917 (10.7)	170 (8.4)	453 (7.9)	1268 (10.9)	898 (11.8)	635 (13.3)	310 (12.3)	183 (8.5)	
Surgical, noncardiac	4433 (12.1)	192 (9.5)	617 (10.7)	1390 (11.9)	996 (13.1)	659 (13.8)	319 (12.6)	260 (12.1)	
Obstetric	93 (0.3)	9 (0.4)	10 (0.2)	39 (0.3)	19 (0.2)	11 (0.2)	3 (0.1)	2 (0.1)	
Trauma	1559 (4.3)	46 (2.3)	103 (1.8)	291 (2.5)	341 (4.5)	320 (6.7)	218 (8.6)	240 (11.2)	
Interventions in place during arrest, n (%)									
Mechanical ventilation	8123 (22.2)	480 (23.7)	953 (16.5)	2084 (17.9)	1869 (24.5)	1335 (27.9)	756 (30.0)	646 (30.0)	<0.001
Vasopressor use	7055 (19.3)	451 (22.3)	901 (15.6)	1994 (17.1)	1571 (20.6)	1061 (22.2)	609 (24.1)	468 (21.8)	<0.001
Dialysis	1088 (3.0)	85 (4.2)	213 (3.7)	359 (3.1)	229 (3.0)	106 (2.2)	57 (2.3)	39 (1.8)	<0.001

Characteristics are shown for patients surviving at least 24 hours after ROSC. IQR indicates interquartile range; and ROSC, return of spontaneous circulation.

The median maximum temperature during the first 24 hours after ROSC was 37.2 °C (interquartile range, 36.8–37.8 °C) (Figure 2). There were 2023 (5.5%) patients in the <36.3 °C temperature group, 5770 (15.8%) in the 36.3 to <36.8 °C group, 11 637 (31.9%) in the 36.8 to <37.3 °C group, 7618 (20.9%) in the 37.3 to <37.8 °C group, 4790 (13.1%) in the 37.8 to <38.3 °C group, 2524 (6.9%) in the 38.3 to <38.8 °C group, and 2152 (5.9%) in the ≥38.8 °C group. Table 1 compares patient characteristics between different maximum temperature ranges after ROSC. Patients in the higher temperature range groups were younger, more likely to have respiratory insufficiency, acute stroke, major trauma, and an initial nonshockable arrest rhythm, and be on mechanical ventilation and continuous intravenous vasopressors at the time of cardiac arrest. Patients in the lower temperature groups were older and more likely to have renal insufficiency.

**Figure 2:**
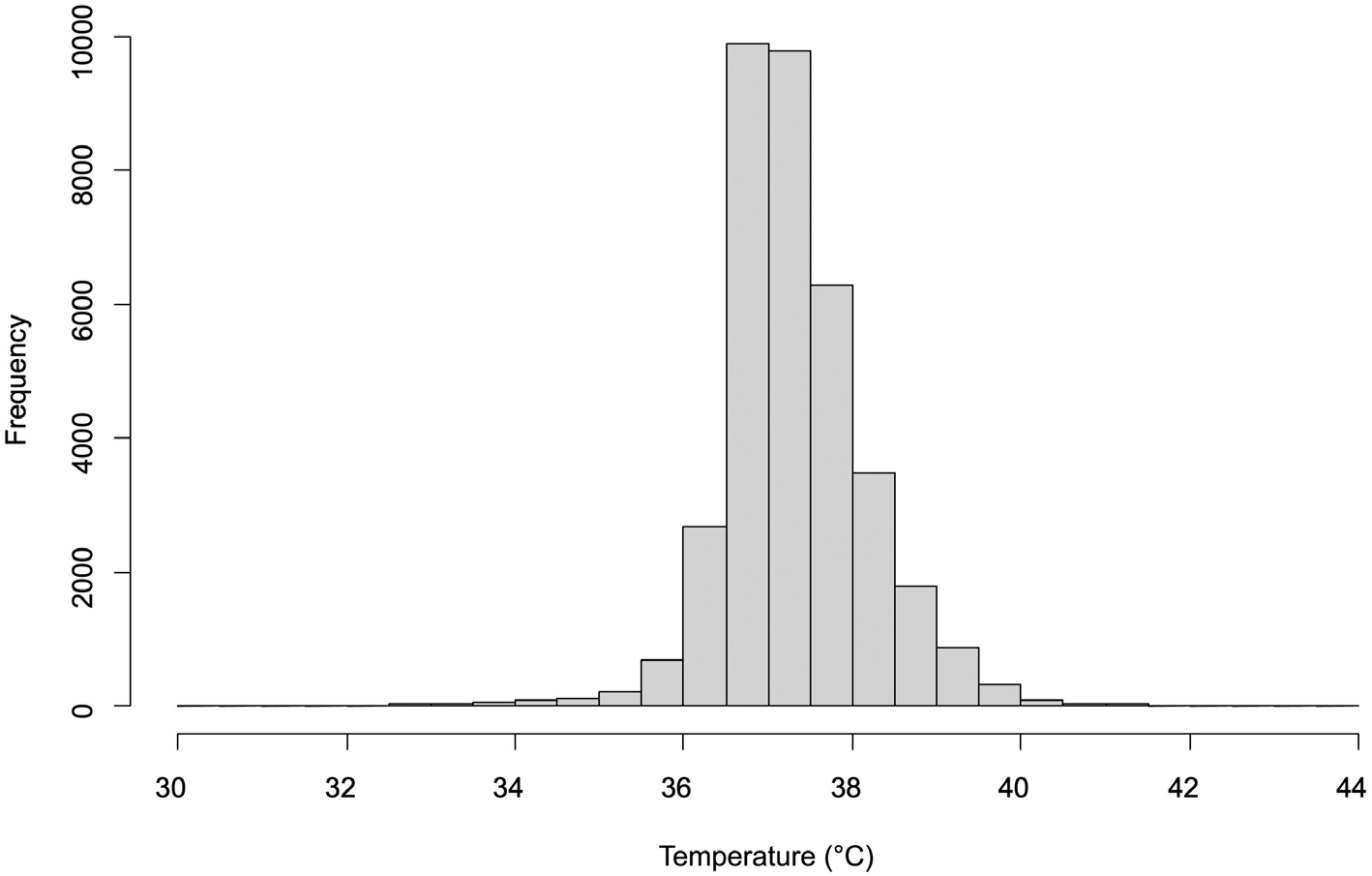
Distribution of maximum temperature during the first 24 hours after ROSC. Shows the distribution of maximum temperature (*x* axis) and the corresponding number of participants (*y* axis). ROSC indicates return of spontaneous circulation.

### Survival Outcomes

Overall, 24 226 (66.3%) patients with IHCA with ROSC survived to hospital discharge. Unadjusted rates of survival to discharge were highest (72.7%) for patients in the reference group (36.8 to <37.3 °C). After multivariable adjustment, an inverted U‐shaped relationship was observed between survival to hospital discharge and maximum temperature during the 24 hours after ROSC (Figure 3). Compared with the reference group, all other temperature groups had lower rates of survival to discharge (all *P*<0.001), especially those in the lowest (<36.3 °C, 48.4%; adjusted odds ratio [OR], 0.42 [95% CI, 0.37–0.46]) and highest (≥38.8 °C, 51.5%; adjusted OR, 0.40 [95% CI, 0.36–0.44]) temperature range (Table 2). Results were similar when the cohort was restricted to the 20 996 patients with at least 5 minutes of resuscitation before achieving ROSC (Table S2).

**Figure 3:**
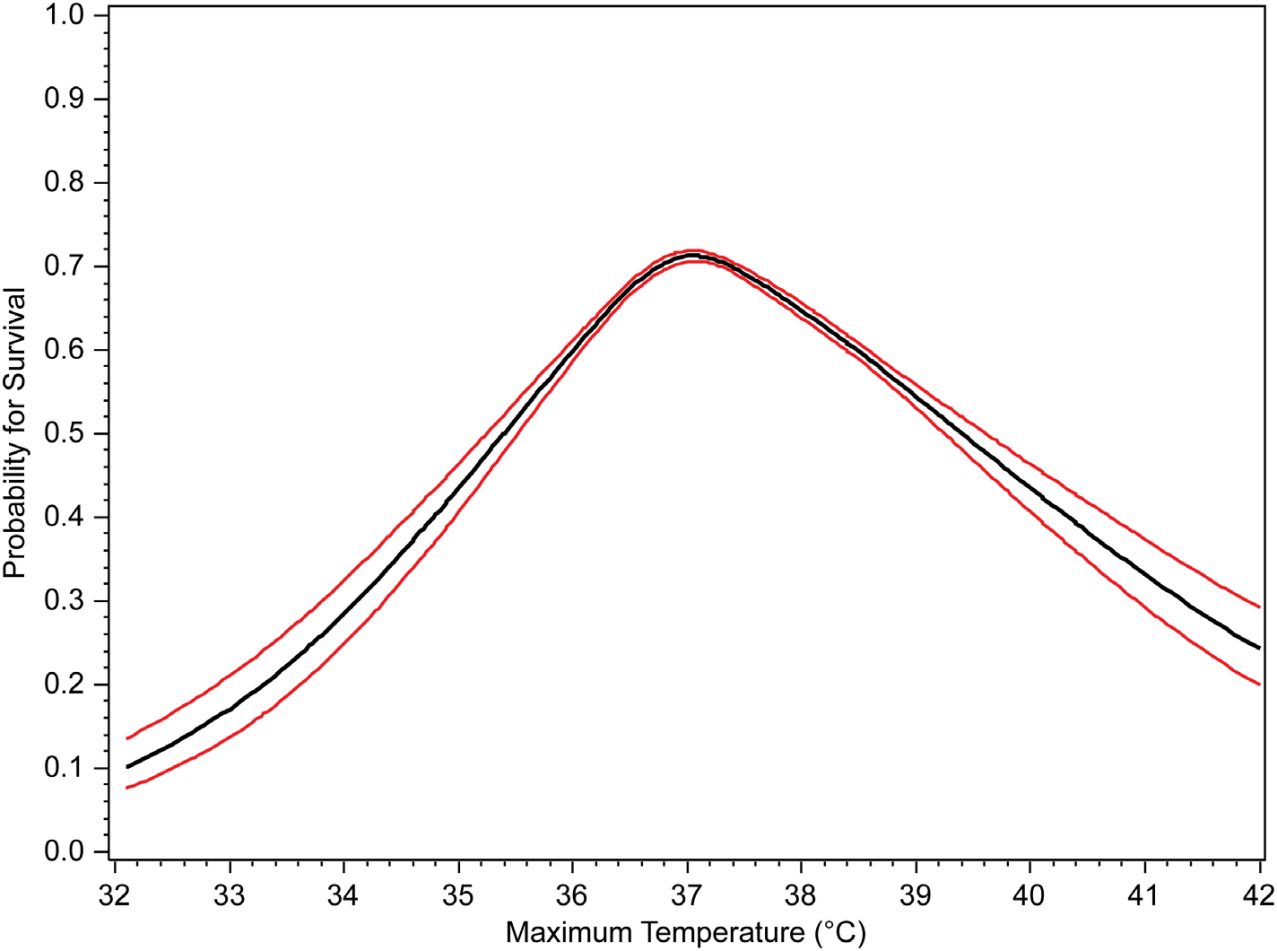
Predicted probability of survival to discharge by maximum temperature during the first 24 hours after ROSC. Using estimates from the multivariable model, the graph shows the predicted probability and 95% CI of survival to discharge (*y* axis) by patients' maximum temperature (*x* axis) among those surviving at least 24 hours after ROSC. The black line indicates the probability of survival; red lines indicate 95% CIs. ROSC indicates return of spontaneous circulation.

**Table 2:** Survival to Discharge by Maximum Temperature During the First 24 Hours After ROSC

Maximum temperature, °C	Survived to discharge, n/N (%)	Unadjusted OR (95% CI)	*P* value	Adjusted OR* (95% CI)	*P* value
<36.3	980/2023 (48.4)	0.36 (0.33–0.4)	<0.0001	0.42 (0.37–0.46)	<0.0001
36.3 to <36.8	3885/5770 (67.3)	0.78 (0.73–0.84)	<0.0001	0.81 (0.75–0.87)	<0.0001
36.8 to <37.3	8457/11637 (72.7)	Reference	Reference	Reference	Reference
37.3 to <37.8	5199/7618 (68.2)	0.82 (0.77–0.87)	<0.0001	0.86 (0.80–0.92)	<0.0001
37.8 to <38.3	3066/4790 (64.0)	0.68 (0.63–0.73)	<0.0001	0.70 (0.65–0.76)	<0.0001
38.3 to <38.8	1531/2524 (60.7)	0.59 (0.54–0.64)	<0.0001	0.60 (0.54–0.66)	<0.0001
≥38.8	1108/2152 (51.5)	0.41 (0.37–0.45)	<0.0001	0.40 (0.36–0.44)	<0.0001

Maximum temperatures are shown for patients surviving at least 24 hours after ROSC. OR indicates odds ratio; and ROSC, return of spontaneous circulation.

* Adjusted models included the following patient‐level covariates as fixed effects: age, sex, race and ethnicity, initial arrest rhythm, arrest location, illness category, comorbid conditions (history of or index admission for myocardial infarction, history of or index admission for heart failure, diabetes, renal insufficiency, hepatic insufficiency, respiratory insufficiency, hypotension, acute stroke, baseline central nervous system depression, metastatic or hematologic malignancy, and metabolic or electrolyte abnormalities), and interventions in place at the time of cardiac arrest (mechanical ventilation, hemodialysis, and continuous intravenous vasopressor use).

A total of 20 818 (57.0%) patients had favorable neurological survival. Unadjusted rates of favorable neurological survival to discharge were similarly highest (64.4%) for patients in the reference temperature group. After multivariable adjustment, a similar inverted U‐shaped relationship was also found between postresuscitation temperature and favorable neurological survival, with the lowest rates of favorable neurological survival among those in the lowest (<36.3 °C, 38.4%; adjusted OR, 0.38 [95% CI, 0.35–0.43]) and highest (≥38.8 °C, 40.2%; adjusted OR, 0.38 [95% CI, 0.34–0.42]) temperature groups (Table 3). Results were similar when the cohort was restricted to patients with at least 5 minutes of resuscitation before achieving ROSC (Table S3).

**Table 3:** Favorable Neurological Survival by Maximum Temperature During the First 24 Hours After ROSC

Maximum temperature, °C	Favorable neurological survival, n/N (%)	Unadjusted OR (95% CI)	*P* value	Adjusted OR* (95% CI)	*P* value
<36.3	776/2023 (38.4)	0.34 (0.31–0.38)	<0.0001	0.38 (0.35–0.43)	<0.0001
36.3 to <36.8	3357/5770 (58.2)	0.77 (0.72–0.82)	<0.0001	0.79 (0.74–0.85)	<0.0001
36.8 to <37.3	7500/11637 (64.4)	Reference	Reference	Reference	Reference
37.3 to <37.8	4453/7618 (58.5)	0.78 (0.73–0.82)	<0.0001	0.83 (0.78–0.89)	<0.0001
37.8 to <38.3	2603/4790 (54.3)	0.66 (0.61–0.71)	<0.0001	0.70 (0.65–0.76)	<0.0001
38.3 to <38.8	1264/2524 (50.1)	0.56 (0.51–0.61)	<0.0001	0.58 (0.53–0.64)	<0.0001
≥38.8	865/2152 (40.2)	0.37 (0.34–0.41)	<0.0001	0.38 (0.34–0.42)	<0.0001

Survival outcomes are reported for patients surviving at least 24 hours after ROSC. OR indicates odds ratio; and ROSC, return of spontaneous circulation.

* Adjusted models included the following patient‐level covariates as fixed effects: age, sex, race and ethnicity, initial arrest rhythm, arrest location, illness category, comorbid conditions (history of or index admission for myocardial infarction, history of or index admission for heart failure, diabetes, renal insufficiency, hepatic insufficiency, respiratory insufficiency, hypotension, acute stroke, baseline central nervous system depression, metastatic or hematologic malignancy, and metabolic or electrolyte abnormalities), and interventions in place at the time of cardiac arrest (mechanical ventilation, hemodialysis, and continuous intravenous vasopressor use).

## Discussion

Given the limited evidence on postresuscitation temperature and survival after IHCA, and using a large national registry, this study is the first to characterize the association between maximum temperature within 24 hours of resuscitation and survival for IHCA. We observed an inverted U‐shaped relationship between maximum temperature during the first 24 hours after ROSC and survival outcomes. Patients with a maximum temperature between 36.8 and <37.3 °C had the highest rates of survival to discharge and favorable neurological survival, whereas those with maximum temperatures in the lowest and highest temperature ranges had the worst outcomes. These findings provide the first description of the natural history of postresuscitation temperature in IHCA and suggest that normothermia is associated with the highest survival compared with higher or lower temperature ranges.

One prior study on IHCA using GWTG–Resuscitation during 2005 through 2006 reported that patients with hypothermia (<36.0 °C) and hyperthermia (≥38 °C) had lower rates of survival to hospital discharge and those with hyperthermia also had lower rates of favorable neurological survival.^15^ However, that study was relatively small (3426 patients), preceded current guidelines of fever avoidance, and did not exclude potential causes of hyperthermia such as sepsis or pneumonia. Otherwise, data on postresuscitation temperature and survival after cardiac arrest is based on patients with OHCA. In a prospective study of 151 patients with OHCA, the risk of unfavorable neurological recovery doubled with each 1 °C above 37 °C (OR, 2.26 [95% CI, 1.24–4.12]).^3^ Furthermore, in 2 small randomized trials of therapeutic hypothermia for OHCA, patients randomized to usual care were more likely to have fever and had lower rates of survival than patients randomized to the hypothermia arm.^4^, ^5^

This study differs from early studies of OHCA by providing novel information on patients with IHCA and extends prior literature in several ways. Using a larger, more representative sample, we compared survival odds across discrete 0.5 °C temperature intervals, rather than relying on predefined cut points or simple fever versus no fever comparisons. Moreover, our comparisons of temperature ranges were risk‐adjusted, ensuring similar patient case mix in the estimates of effect, and we excluded patients more prone to fever to reduce potential bias. Finally, the findings were robust even when restricted to patients with at least 5 minutes of resuscitation before achieving ROSC.

Our findings provide a more detailed understanding of the natural history between postresuscitation temperature and survival for patients with IHCA without overt sepsis who were not treated with active hypothermia. A maximum temperature in the normothermic range was associated with the highest likelihood of survival, whereas temperatures at both the high and low extremes were associated with worse outcomes. Patients in the lower and higher temperature groups likely had temperature dysregulation because of underlying illness severity or anoxic brain injury, which has been described for OHCA.^16^ Consistent with prior studies supporting fever avoidance, these results reinforce the importance of preventing hyperthermia. However, whether actively correcting spontaneous hypothermia or targeting a specific normothermic range improves survival remains unknown. Further studies testing this hypothesis, including in randomized trials, are needed to examine whether such a postresuscitation temperature management strategy is efficacious.

This study should be interpreted in the context of several limitations. First, GWTG–Resuscitation captures only the maximum temperature in the first 24 hours after ROSC and does not quantify the timing within the first 24 hours nor the duration of time a patient is in a given temperature range, so the findings need to be interpreted in this context. Second, temperature information on patients beyond the 24‐hour period was not available nor was information on the modality of temperature assessment (eg, oral, axillary, rectal), which could lead to some misclassification of maximum temperatures. Third, approximately one third of otherwise eligible patients were excluded because of missing temperature data. Although included and excluded patients were generally similar with most absolute SMDs <0.10 and none exceeding 0.20, some degree of selection bias cannot be completely excluded. Fourth, although patients with documented causes for developing fever (eg, sepsis, pneumonia, and COVID‐19) were excluded, patients with prearrest fever due to undiagnosed infections would have been included in the study cohort. Additionally, despite multivariable hierarchical adjustment for numerous patient demographic‐, arrest‐, and intervention‐related covariates, residual confounding from unmeasured or incompletely measured variables cannot be excluded. Fifth, participation in the GWTG–Resuscitation registry is voluntary; therefore, the results may not be generalizable to other hospitals, although there is no reason to expect this relationship to differ in nonparticipating hospitals. Sixth, the registry lacks information on neurological assessments or variables to determine the extent of neurological injury after a resuscitation event to correlate with observed temperatures. Finally, the registry does not include information on whether and when a hospital implemented protocols for postresuscitation temperature management or on cause of death.

## Conclusions

Among patients successfully resuscitated from IHCA and not treated with therapeutic hypothermia, the highest survival was observed in those with a maximum temperature between 36.8 and <37.3 °C during the first 24 hours after ROSC compared with those with higher or lower postresuscitation temperature ranges.

## Sources of Funding

Drs Cantu‐Martinez, Abdel Jawad and Girard are supported by the National Heart, Lung, and Blood Institute (T32H110837). Dr Chan is supported by the National Heart, Lung, and Blood Institute (R01HL160734 and R01HL178638). The content is solely the responsibility of the authors and does not necessarily represent the official views of the National Institutes of Health.

## Disclosures

K. F. Kennedy reports no disclosures. Dr Ikemura reports no disclosures. Dr Chan receives funding from the American Heart Association and is a consultant for Optum Rx and serves as a member of the American Heart Association Get With The Guidelines–Resuscitation Research Task Force. In accordance with registry policies, they recused themselves from scoring and all decision making related to the approval of this study's proposal. Dr Spertus provides consulting services on patient‐reported outcomes and evidence evaluation to 4DT Medical, Alnylam, AstraZeneca, Bayer, Bristol Meyers Squibb, Cytokinetics, Imbria, Janssen, Merck, Terumo, and UnitedHealthcare; holds research grants from Bristol Meyers Squibb and Janssen; owns the copyright to the Seattle Angina Questionnaire, Kansas City Cardiomyopathy Questionnaire, and Peripheral Artery Questionnaire; and serves on the Board of Directors for Blue Cross Blue Shield of Kansas City.

## Supporting information

STROBE Statement Checklist S1

Tables S1–S3

## Appendix A

The Get With The Guidelines programs are provided by the American Heart Association.

American Heart Association's Get With The Guidelines–Resuscitation Adult Research Task Force members: Anne Grossestreuer, PhD; Ari Moskowitz, MD, MPH; Joseph Ornato, MD, FACP, FACC, FACEP; Matthew Churpek, MD, MPH, PhD; Paul Chan, MD, MSc; Saket Girotra, MD, SM; Sarah Perman, MD, MSCE.
