# Supplementary material for: Association Between Maximum Temperature 24 Hours After Resuscitation and Survival for In‐Hospital Cardiac Arrest
Source: J Am Heart Assoc. 2026 Aug 20;15(17):e048549. doi: 10.1161/JAHA.125.048549 (PMC13695831; doi:10.1161/JAHA.125.048549)
Supplement: Supplementary file 1 [file JAH3-15-e048549-s001.pdf]

Supplemental Material

Table S1. Comparison of Baseline Characteristics Between Patients Included in the Final Cohort and Patients Excluded Because of Missing Temperature Data.

CHARACTERISTICS	INCLUDED n = 36514	EXCLUDED* n = 30,324	SMD
**DEMOGRAPHICS**			
Age, year, mean ± SD	64.2 ± 16.6	64.6 ± 35.8	0.01
Sex			0.01
Male	22583 (61.8%)	18575 (61.3%)	
Female	13931 (38.2%)	11749 (38.7%)	
Race			0.08
White	25330 (69.4%)	20171 (66.5%)	
Black	7681 (21.0%)	6713 (22.1%)	
Asian non-Hispanic	792 (2.2%)	795 (2.6%)	
Other or unknown	2709 (7.4%)	2640 (8.7%)	
Hispanic ethnicity	2225 (6.1%)	2239 (7.4%)	
**PRE-EXISTING CONDITIONS**			
Respiratory insufficiency	14621 (40.0%)	11664 (38.5%)	0.03
Diabetes mellitus	13995 (38.3%)	10694 (35.3%)	0.06
Renal insufficiency	11123 (30.5%)	9781 (32.3%)	0.04
Hypotension	8977 (24.6%)	5687 (18.8%)	0.14
Metabolic or electrolyte abnormality	7311 (20.0%)	5685 (18.7%)	0.03
Heart failure this admission	5647 (15.5%)	3625 (12.0%)	0.10
History of prior heart failure	10022 (27.4%)	6875 (22.7%)	0.11
Myocardial infarction this admission	6299 (17.3%)	3945 (13.0%)	0.12
History of myocardial infarction	5956 (16.3%)	3864 (12.7%)	0.10
Acute CNS non-stroke event	4372 (12.0%)	2145 (7.1%)	0.17
Acute stroke	1537 (4.2%)	1300 (4.3%)	0.01
Baseline depression in CNS function	2505 (6.9%)	1549 (5.1%)	0.07
Metastatic or hematologic malignancy	3163 (8.7%)	2512 (8.3%)	0.01
Hepatic insufficiency	2579 (7.1%)	2189 (7.2%)	0.01
Major trauma	1996 (5.5%)	1320 (4.4%)	0.05
**CARDIAC ARREST CHARACTERISTICS**			
Initial Arrest Rhythm			0.04
Asystole	8248 (22.6%)	6956 (22.9%)	
Pulsless electrical activity	18416 (50.4%)	15728 (51.9%)	
Ventricular fibrillation	5361 (14.7%)	4216 (13.9%)	
Pulseless ventricular tachycardia	4489 (12.3%)	3424 (11.3%)	
Location of Cardiac Arrest			0.13

Intensive Care Unit	13936 (38.2%)	12281 (40.5%)	
Telemetry	6416 (17.6%)	4594 (15.2%)	
Non Monitored	4955 (13.6%)	4977 (16.4%)	
Emergency room	4983 (13.6%)	3965 (13.1%)	
Procedural	5404 (14.8%)	3668 (12.1%)	
Other	818 (2.2%)	835 (2.8%)	
Illness Category			0.11
Medical-Cardiac	13547 (37.1%)	12476 (41.1%)	
Medical-Noncardiac	12905 (35.3%)	10847 (35.8%)	
Surgical-Cardiac	3917 (10.7%)	2846 (9.4%)	
Surgical-Noncardiac	4433 (12.1%)	3057 (10.1%)	
Obstetric	93 (0.3%)	72 (0.2%)	
Trauma	1559 (4.3%)	976 (3.2%)	
**INTERVENTIONS IN PLACE DURING ARREST**			
Mechanical ventilation	8123 (22.2%)	7051 (23.3%)	0.02
Vasopressor use	7055 (19.3%)	5426 (17.9%)	0.04
Dialysis	1088 (3.0%)	741 (2.4%)	0.03

Abbreviations: CNS, central nervous system; SD, standard deviation; SMD, standardized mean difference.

*Excluded patients included those with extreme temperatures (<30°C or >44°C; n=153), those treated at hospitals with >25% missing temperature data (n=27,257), and those with missing maximum temperature data (n=2,914).

Table S2. Survival to Discharge by Maximum Temperature During the First 24 Hours After ROSC in Patients with ≥ 5 Minutes of Resuscitation.

Maximum Temperature	Survived to Discharge	Unadjusted OR (95% CI)	P-value	Adjusted OR* (95% CI)	P-value
<36.3°C	625/1388 (45.0%)	0.45 (0.4–0.51)	<.0001	0.49 (0.43–0.56)	<.0001
36.3 to <36.8°C	1869/3118 (59.9%)	0.82 (0.75–0.9)	<.0001	0.84 (0.76–0.92)	<0.001
36.8 to <37.3°C	3942/6102 (64.6%)	Reference	Reference	Reference	Reference
37.3 to <37.8°C	2800/4494 (62.3%)	0.92 (0.84–0.99)	0.0326	0.92 (0.84–1.00)	0.04
37.8 to <38.3°C	1754/2947 (59.5%)	0.81 (0.74–0.89)	<.0001	0.79 (0.72–0.87)	<.0001
38.3 to <38.8°C	851/1528 (55.7%)	0.69 (0.62–0.78)	<.0001	0.67 (0.59–0.75)	<.0001
≥38.8°C	656/1419 (46.2%)	0.48 (0.42–0.54)	<.0001	0.43 (0.38–0.49)	<.0001

Abbreviations: CI, confidence interval; OR, odds ratio; ROSC, return of spontaneous circulation.
*Adjusted models included the following patient-level covariates as fixed effects: age, sex, race/ethnicity, initial arrest rhythm, arrest location, illness category, comorbid conditions (history of or index admission for myocardial infarction, history of or index admission for heart failure, diabetes mellitus, renal insufficiency, hepatic insufficiency, respiratory insufficiency, hypotension, acute stroke, baseline central nervous system depression, metastatic or hematologic malignancy, and metabolic or electrolyte abnormalities), and interventions in place at the time of cardiac arrest (mechanical ventilation, hemodialysis, and continuous intravenous vasopressor use).

Table S3. Favorable Neurological Survival by Maximum Temperature During the First 24 Hours After ROSC in Patients with ≥ 5 Minutes of Resuscitation.

Maximum Temperature	Favorable Neurological Survival	Unadjusted OR (95% CI)	P-value	Adjusted OR* (95% CI)	P-value
<36.3°C	485/1388 (34.9%)	0.42 (0.37–0.47)	<.0001	0.45 (0.40–0.51)	<.0001
36.3 to <36.8°C	1546/3118 (49.6%)	0.78 (0.72–0.85)	<.0001	0.80 (0.73–0.87)	<.0001
36.8 to <37.3°C	3380/6102 (55.4%)	Reference	Reference	Reference	Reference
37.3 to <37.8°C	2333/4494 (51.9%)	0.88 (0.81–0.95)	0.001	0.89 (0.82–0.96)	0.004
37.8 to <38.3°C	1429/2947 (48.5%)	0.76 (0.69–0.83)	<.0001	0.75 (0.68–0.82)	<.0001
38.3 to <38.8°C	679/1528 (44.4%)	0.65 (0.58–0.72)	<.0001	0.64 (0.56–0.72)	<.0001
≥38.8°C	518/1419 (36.5%)	0.47 (0.41–0.53)	<.0001	0.44 (0.38–0.50)	<.0001

Abbreviations: CI, confidence interval; OR, odds ratio; ROSC, return of spontaneous circulation.
*Adjusted models included the following patient-level covariates as fixed effects: age, sex, race/ethnicity, initial arrest rhythm, arrest location, illness category, comorbid conditions (history of or index admission for myocardial infarction, history of or index admission for heart failure, diabetes mellitus, renal insufficiency, hepatic insufficiency, respiratory insufficiency, hypotension, acute stroke, baseline central nervous system depression, metastatic or hematologic malignancy, and metabolic or electrolyte abnormalities), and interventions in place at the time of cardiac arrest (mechanical ventilation, hemodialysis, and continuous intravenous vasopressor use).
